# Complete genome sequence of “*Thioalkalivibrio sulfidophilus*” HL-EbGr7

**DOI:** 10.4056/sigs.1483693

**Published:** 2011-02-14

**Authors:** Gerard Muyzer, Dimitry Yu Sorokin, Konstantinos Mavromatis, Alla Lapidus, Alicia Clum, Natalia Ivanova, Amrita Pati, Patrick d'Haeseleer, Tanja Woyke, Nikos C. Kyrpides

**Affiliations:** 1 Department of Biotechnology, Delft University of Technology, Delft, The Netherlands; 2 Winogradsky Institute of Microbiology, Russian Academy of Sciences, Moscow, Russia; 3 Joint Genome Institute, Walnut Creek, California, USA; 4 Joint Bioenergy Institute, California, USA

**Keywords:** haloalkaliphilic, sulfide, thiosulfate, sulfur-oxidizing bacteria (SOB)

## Abstract

“*Thioalkalivibrio sulfidophilus*” HL-EbGr7 is an obligately chemolithoautotrophic, haloalkaliphilic sulfur-oxidizing bacterium (SOB) belonging to the *Gammaproteobacteria*. The strain was found to predominate a full-scale bioreactor, removing sulfide from biogas. Here we report the complete genome sequence of strain HL-EbGr7 and its annotation. The genome was sequenced within the Joint Genome Institute Community Sequencing Program, because of its relevance to the sustainable removal of sulfide from bio- and industrial waste gases.

## Introduction

“*Thioalkalivibrio sulfidophilus*” HL-EbGr7 is an obligately chemolithoautotrophic SOB using CO_2_ as a carbon source and reduced inorganic sulfur compounds as an energy source. It belongs to the genus *Thioalkalivibrio*. This genus is characterized by obligate haloalkaliphily and forms a monophyletic group within the family *Ectothiorhodospiraceae*. The genus currently includes nine validly described species [1] and many yet uncharacterized isolates [2,3]. The members are slow growing and well-adapted to hypersaline (up to salt saturation) and alkaline (up to pH 10.5) conditions. They can oxidize sulfide, thiosulfate, elemental sulfur, sulfite and polythionates (see Table 1 for summary). Moreover, some species can reduce nitrate, nitrite or nitrous oxide [17,18] or utilize thiocyanate (SCN^-^) as an energy and nitrogen source [19,20]. Genetic diversity analysis of 85 *Thioalkalivibrio* strains isolated from different soda lakes located in Mongolia, Kenya, California, Egypt and south Siberia, indicated a high genetic diversity and an endemic character, i.e., the majority of the genotypes (85.9%) were found to be unique to one region [15].

**Table 1:** Classification and general features of “*Thioalkalivibrio sulfidophilus*” strain HL-EbGR7 according to the MIGS recommendations [4].

**MIGS ID**	**Property**	**Term**	**Evidence code**
	Current classification	Domain *Bacteria*	TAS [5]
		Phylum *Proteobacteria*	TAS [6]
		Class *Gammaproteobacteria*	TAS [7,8]
		Order *Chromatiales*	TAS [7,9]
		Family *Ectothiorhodospiraceae*	TAS [10]
		Genus *Thioalkalivibrio*	TAS [11-13]
		Species “*Thioalkalivibrio sulfidophilus*” HL-EbGR7	NAS
	Gram stain	negative	TAS [2]
	Cell shape	rod-shaped	TAS [2]
	Motility	motile	TAS [2]
	Sporulation	non-sporulating	TAS [2]
	Temperature range	Mesophile	TAS [2]
	Optimum temperature	34	TAS [2]
MIGS-6.3	Salinity range	0.2-1.5M Na^+^ (opt.0.4 M)	TAS [2]
MIGS-22	Oxygen requirement	microaerophilic	TAS [2]
	Carbon source	HCO_3_^-^	TAS [2]
	Energy source	Sulfide/polysulfide, thiosulfate, sulfur	TAS [2]
MIGS-6	Habitat	Alkaline bioreactors; soda lakes	TAS [2]
MIGS-15	Biotic relationship	free-living	TAS [2]
MIGS-14	Pathogenicity	none	NAS
	Biosafety level	1	TAS [14]
	Isolation	Thiopaq bioreactor	TAS [15]
MIGS-4	Geographic location	Eerbeek, The Netherlands	TAS [15]
MIGS-5	Sample collection time	2005	NAS
MIGS-4.1	Latitude	52.11	TAS [16]
MIGS-4.2	Longitude	6.07	TAS [16]
MIGS-4.3	Depth	Not applicable	
MIGS-4.4	Altitude	Sea level	NAS

Evidence codes - IDA: Inferred from Direct Assay (first time in publication); TAS: Traceable Author Statement (i.e., a direct report exists in the literature); NAS: Non-traceable Author Statement (i.e., not directly observed for the living, isolated sample, but based on a generally accepted property for the species, or anecdotal evidence). These evidence codes are from of the Gene Ontology project. If the evidence code is IDA, then the property was directly observed by one of the authors or an expert mentioned in the acknowledgements.

Apart from their role in the sulfur cycle of soda lakes, *Thioalkalivibrio* species also play a key role in the sustainable removal of sulfide from wastewater and gas streams. In this so-called ‘Thiopaq-process’, hydrogen sulfide is stripped from the gas phase into an alkaline solution, which is subsequently transferred to a bioreactor where *Thioalkalivibrio* oxidizes HS^-^ almost exclusively to elemental sulfur at a low red-ox potential [21]. Removal of toxic sulfide is needed, not only for a clean and healthy environment, but also to protect gas turbines from corrosion. In contrast to chemical desulfurization processes, such as the ‘Claus-process’, biological removal is cheaper, cleaner and more sustainable, as the produced hydrophilic bio-sulfur is a better fertilizer and fungicide than the chemically produced crystalline hydrophobic sulfur.

To get insight into the molecular mechanism by which *Thioalkalivibrio* strains adapt to haloalkaline conditions (i.e., pH 10 and up to 4 M of Na^+^) identification of the genes that are involved in these adaptations is needed. The most important issues are sulfide specialization, carbon assimilation at high pH and bioenergetic adaptation to high salt/high pH. In addition, information on the genome might help in optimizing the sulfur removal process. Here we present a summary classification and a set of features for “*T. sulfidophilus*” HL-EbGr7, together with the description of the genomic sequencing and annotation.

## Classification and features

**Figure 1:**
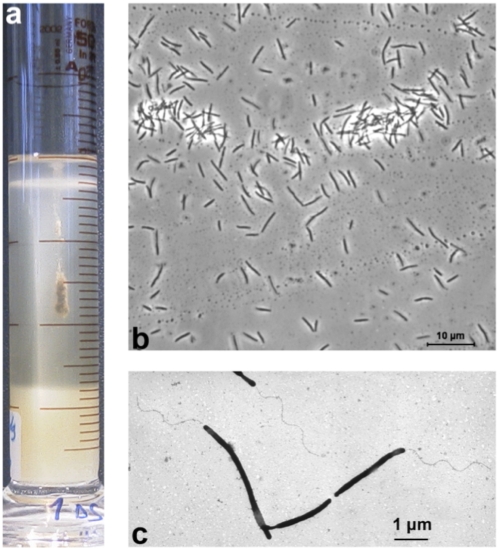
**a**, Enrichment of “*Thioalkalivibrio sulfidophilus*” HL-EbGr7 in a gradient culture, whereby sulfide is diffusing from an agarose plug in the bottom of the cylinder and O_2_ from the top. The bacterial cells accumulate in a dense band at the most favorable sulfide and oxygen concentration. **b**, phase-contrast microphotograph; **c**, electron microscopy microphotograph of a total cell preparation contrasted with phosphotungstic acid.

**Figure 2:**
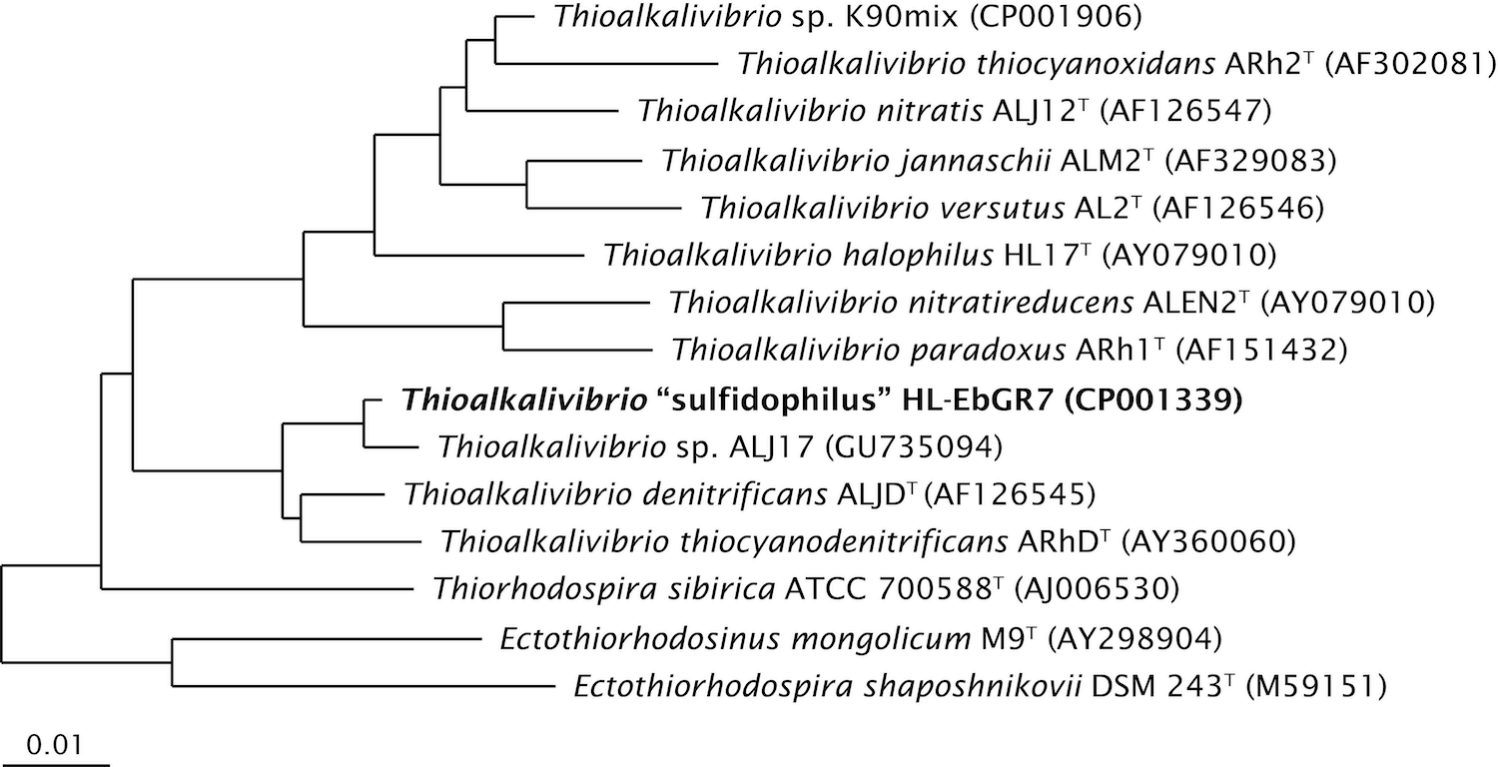
Phylogenetic tree based on 16S rRNA sequences showing the phylogenetic position of “*Thioalkalivibrio sulfidophilus*” HL-EbGr7. The sequence was aligned to sequences stored in the SILVA database using the SINA Webaligner [23]. Subsequently, the aligned sequences were imported into ARB [24], and a neighbor joining tree was constructed. Sequences of members from the *Alphaproteobacteria* were used as an outgroup, but were pruned from the tree. The scale bar indicates 1% sequence difference.

“*T. sulfidophilus*” HL-EbGr7 was isolated from a full-scale Thiopaq bioreactor in the Netherlands used to remove H_2_S from biogas [21]. The reactor biomass had a very peculiar property, which made it different from the usual SOB biomass, i.e., an almost complete sulfide specialization and no thiosulfate-oxidizing activity. This was probably the result of a very low red-ox potential at which the reactor was operated. Therefore, the dominant SOB could originally be enriched only with sulfide as substrate in cylinders with agarose-stabilized medium containing opposing gradients of oxygen and sulfide [Figure 1a, 22]. Subsequently, the strain was purified using serial dilutions in gradient cultures and finally from a colony on solid medium with sulfide at micro-oxic conditions. It has rod-shaped, elongated cells with a polar flagellum (Figure 1b and c). The strain is obligately alkaliphilic with a pH optimum of 9.5. It can tolerate a salinity of 1.5 M (optimum at 0.4 M) of total sodium, sulfide concentrations up to 5 mM and a temperature up to 40°C. It utilizes ammonium and urea, but not nitrate or nitrite, as a N-source. On the basis of 16S rRNA gene sequencing the strain belongs to the *Gammaproteobacteria* with *Thioalkalivibrio denitrificans* as the closest, described species (Figure 2). Despite this relation, strain HL-EbGr7 cannot grow anaerobically with NOx. Both phylogeny and specific physiology indicate that this strain represents a novel species within the genus *Thioalkalivibrio* for which a tentative species epithet “sulfidophilus” is proposed.

## Genome sequencing information

### Genome project history

Strain HL-EbGr7 was selected for sequencing in the 2007 Joint Genome Institute Community Sequencing Program, because of its relevance to bioremediation. A summary of the project information is presented in Table 2. The complete genome sequence was finished in December 2008. The GenBank accession number for the project is NC_011901. The genome project is listed in the Genome OnLine Database (GOLD) [25] as project Gc00934. Sequencing was carried out at the Joint Genome Institute (JGI). Finishing was done by JGI-Los Alamos National Laboratory (LANL) and initial automatic annotation by JGI-Oak Ridge National Laboratory (ORNL).

**Table 2:** Genome sequencing project information

**MIGS ID**	**Characteristic**	**Details**
MIGS-28	Libraries used	6kb Sanger and 454 standard libraries
MIGS-29	Sequencing platform	ABI-3730, 454 GS FLX Titanium
MIGS-31.2	Sequencing coverage	8.19 × Sanger, 23.3 × pyrosequence
MIGS-31	Finishing quality	Finished
	Sequencing quality	Less than one error per 50kb
MIGS-30	Assembler	Newbler, PGA
MIGS-32	Gene calling method	Prodigal, GenePRIMP
	GenBank ID	NC_011901
	GenBank date of release	December 29, 2008
	GOLD ID	Gc00934
	NCBI project ID	29177
	IMG Taxon ID	643348585
MIGS-13	Source material identifier	Personal culture collection, Winogradsky Institute of Microbiology, Moscow
	Project relevance	Bioremediation

### Growth conditions and DNA isolation

After a long-term gradual adaptation on mixed substrate medium, the isolate was able to grow solely with thiosulfate at micro-oxic conditions. The medium contained 40 mM thiosulfate as an energy source and a standard sodium carbonate-bicarbonate buffer [2] (Sorokin et al., 2006) at pH 10 and 0.6 M Na^+^. The cells were harvested by centrifugation and stored at -80°C for DNA extraction. Genomic DNA was obtained using phenol-chloroform-isoamylalcohol (PCI) extraction. Briefly, the cell pellet was suspended in a Tris-EDTA buffer at pH 8, and lysed with a mixture of SDS and Proteinase K. The genomic DNA was extracted using PCI and precipitated with ethanol. The pellet was dried under vacuum and subsequently dissolved in water. The quality and quantity of the extracted DNA was evaluated using the DNA Mass Standard Kit provided by the JGI.

### Genome sequencing and assembly

The genome of “*T. sulfidophilus*” HL-EbGr7 was sequenced using a combination of Sanger and 454 sequencing platforms. All general aspects of library construction and sequencing can be found at the JGI website [26]. Pyrosequencing reads were assembled using the Newbler assembler version 1.1.02.15 (Roche). Large Newbler contigs were broken into 7,722 overlapping fragments of 1,000 bp and entered into assembly as pseudo-reads. The sequences were assigned quality scores based on Newbler consensus q-scores with modifications to account for overlap redundancy and adjust inflated q-scores. A hybrid 454/Sanger assembly was made using the Parallel Genome Assembler (PGA). Possible mis-assemblies were corrected and gaps between contigs were closed by editing in Consed, or by custom primer walks of sub-clones or PCR products. A total of 518 Sanger finishing reads were produced to close gaps, to resolve repetitive regions, and to raise the quality of the finished sequence. The error rate of the completed genome sequence is less than 1 in 100,000. Together, the combination of the Sanger and 454 sequencing platforms provided a 31.49-times coverage of the genome. The final assembly contains 32,486 Sanger reads and 390,057 pyrosequencing reads.

### Genome annotation

Genes were identified using Prodigal [27] as part of the Oak Ridge National Laboratory genome annotation pipeline followed by a round of manual curation using the JGI GenePRIMP pipeline [28]. The predicted CDSs were translated and used to search the National Center for Biotechnology Information (NCBI) nonredundant database, UniProt, TIGRFam, Pfam, PRIAM, KEGG, COG, and InterPro, databases. Additional gene prediction analysis and functional annotation were performed within the Integrated Microbial Genomes Expert Review (IMG-ER) platform [29].

## Genome properties

**Figure 3:**
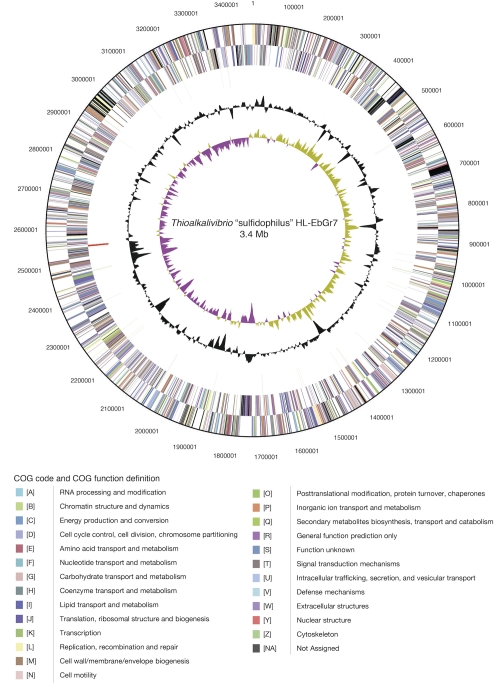
Graphical circular map of the chromosome of “*Thioalkalivibrio sulfidophilus*” HL-EbGr7. From outside to the center: Genes on the forward strand (Colored by COG categories), Genes on the reverse strand (colored by COG categories), RNA genes (tRNAs green, rRNAs red, other RNAs black), GC content, GC skew.

The genome of strain HL-EbGr7 consists of a single circular chromosome (Figure 3) with a size of 3.46 Mbp. The G+C percentage determined from the genome sequence is 65.06%, which is a little higher than the G+C content determined by thermal denaturation (63.5 + 0.5 mol%). There are 3,366 genes of which 3,319 are protein-coding genes and the remaining 47 are RNA genes. 36 pseudogenes were identified, constituting 1.07% of the total number of genes. The properties and statistics of the genome are summarized in Table 3, and genes belonging to COG functional categories are listed in Table 4.

**Table 3:** Genome statistics

**Attribute**	**Value**	**% of Total**
Genome size (bp)	3,464,554	100.00%
DNA coding region (bp)	3,030,998	87.49%
DNA G+C content (bp)	2,254,142	65.06%
Number of replicons	1	
Extrachromosomal elements	0	
Total genes	3366	100.00%
RNA genes	47	1.40%
rRNA operons	3	0.09%
Protein-coding genes	3319	98.06%
Pseudogenes	36	1.07%
Genes in paralog clusters	294	8.73%
Genes assigned to COGs	2512	74.36%
Genes assigned Pfam domains	2653	78.82%
Genes with signal peptides	719	21.36%
CRISPR repeats	2	

**Table 4:** Number of genes associated with the general COG functional categories.

**Code**	**value**	**%age**	**COG category**
E	180	6.39	Amino acid transport and metabolism
G	94	3.34	Carbohydrate transport and metabolism
D	49	1.74	Cell cycle control, cell, division, chromosome partitioning
N	109	3.87	Cell motility
M	199	7.06	Cell wall/membrane/envelope biogenesis
B	2	0.07	Chromatin structure and dynamics
H	139	4.93	Coenzyme transport and metabolism
Z	0	0	Cytoskeleton
V	37	1.31	Defense mechanism
C	184	6.53	Energy production and conversion
W	0	0	Extracellular structures
S	270	9.58	Function unknown
R	310	11.00	General function prediction only
P	152	5.39	Inorganic ion transport and metabolism
U	117	4.15	Intracellular trafficking, secretion, and vesicular transport
I	70	2.48	Lipid transport and metabolism
Y	0	0	Nuclear structure
F	58	2.06	Nucleotide transport and metabolism
O	142	5.04	Posttranslational modification, protein turnover, chaperones
A	2	0.07	RNA processing and modification
L	145	5.15	Replication, recombination and repair
Q	47	1.67	Secondary metabolites biosynthesis, transport and catabolism
T	216	7.67	Signal transduction mechanisms
K	134	4.76	Transcription
J	162	5.75	Translation, ribosomal structure and biogenesis
-	854	25.37	Not in COGs

## Insights from the genome sequence

### Autotrophic growth

One of the major problems of autotrophic growth at high pH is the assimilation of inorganic carbon (Ci); carbon dioxide concentrations are very low and most inorganic carbon is present as HCO_3_^-^ or even as CO_3_^2-^ at pH values of 10 and higher. The latter is not available to the cell, which is the main reason for growth limitation of haloalkaliphilic SOB at pH above 10.5, since their energy generation respiratory system is still active up to pH 11-11.5 [2]. Inside the cells, where Ci assimilation occurs, the pH is around 8.5, which means that HCO_3_^-^ must be taken up as a substrate at an exterior pH of 10. This demands active transport by means of a Na^+^/HCO_3_- symporter, such as StbA, which has been found in the alkaliphilic cyanobacterium *Synechocystis* sp. strain PCC6803 [30]. However, genes encoding StbA have not been detected in strain HLEbGr7. Another means of growth at limited CO_2_ concentrations is the use of a carbon-concentrating mechanism (CCM), which has been described for other autotrophic microorganisms [31]. Part of the CCM is the presence of carboxysomes, in which ribulose-1,5-biphosphate carboxylase/oxygenase (RuBisCO) and carbonic anhydrase, the key enzymes in CO_2_ fixation, are located in close proximity for an efficient carbon fixation [32]. The genome of strain HL-EbGr7 contains the genes for the large (rbcL) and small subunit (rbcS) of RuBisCO form 1Ac, and for the synthesis of a-carboxysomes, including *csoSCA* (formerly know as *csoS3*) encoding a carboxysome shell carbonic anhydrase. The latter is necessary to convert the transported HCO_3_^-^ into CO_2_ – the actual substrate of RuBisCO. In contrast to *Thiomicrospira crunogena,* the genomes of *Thioalkalivibrio* are lacking genes for RuBisCO form 1Aq and form II, which has been confirmed by Tourova *et al*. [33]. Expression studies at different CO_2_ concentrations in the chemolithoautotroph *Hydrogenovibrio marinus* indicated the preferential expression of RuBisCO form 1Ac at low CO_2_ concentrations and RuBisCO form 1Aq and/or form II at higher CO_2_ concentrations [34]. This result indicates that our strain is indeed adapted to low CO_2_ concentrations.

### Sulfur metabolism

**Figure 4:**
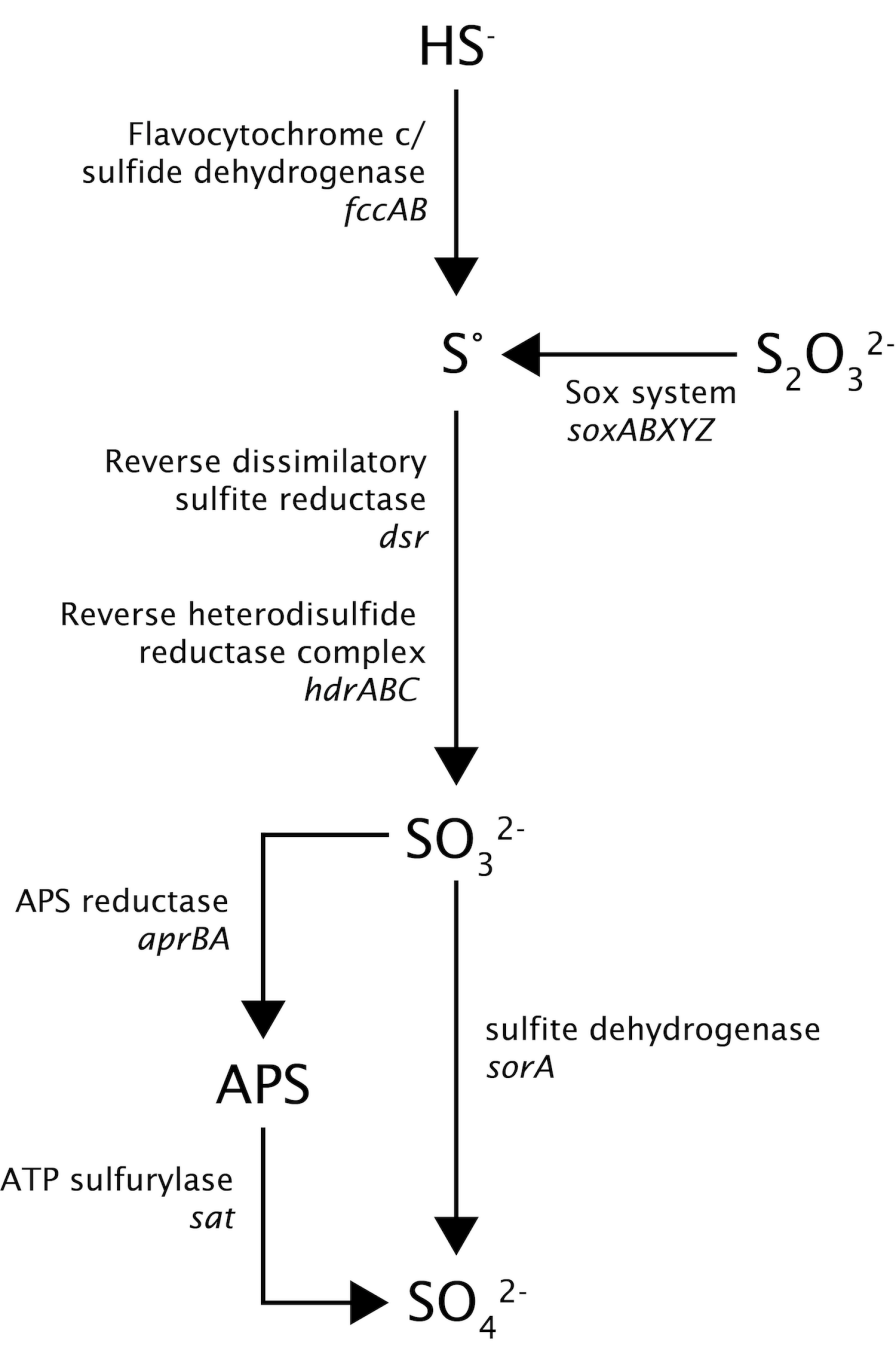
Hypothetical pathways for oxidation of sulfur compounds in “*Thioalkalivibrio sulfidophilus*” HL-EbGr7.

*Thioalkalivibrio* species can oxidize reduced sulfur compounds, such as sulfide and thiosulfate, to elemental sulfur and subsequently to sulfate. However, little is known about the enzymes that are involved in the sulfur metabolism of these organisms. Figure 4 shows the pathway of sulfur metabolism in strain HL-EbGr7 inferred from the genes found in the genome. Sulfide is oxidized by flavocytochrome *c*/sulfide dehydrogenase; both genes, encoding the small subunit A (*fccA*), and the large subunit B (*fccB*) were present in 3 copies. Although sulfide:quinone oxidoreductase (SQR) activity had been found in *Thioalkalivibrio* species, the *sqr* gene could not be detected. A similar result has also been found for *Allochromatium vinosum* [35]. The presence of a truncated Sox cluster consisting of *soxXAYZB,* but lacking *soxCD,* leads to the formation of elemental sulfur as an intermediate [35], and also gives the organism the possibility to oxidize the sulfane moiety of thiosulfate, which has been confirmed by culture studies. The soxXA genes were present in 4 copies; the sox YZ genes in 2 copies. Subsequently, elemental sulfur is oxidized to sulfite by a reversed dissimilatory sulfite reductase pathway, consisting of *dsrABEFHCMKLJOPNR.* In addition, we found genes (*hdrABC*) encoding a heterodisulfide reductase complex, which was highly similar to that found in *Acidithiobacillus ferrooxidans* and was hypothesized to work in reverse [36]. Sulfite can be oxidized to sulfate, either directly by sulfite dehydrogenase (*sorA*) or indirectly via adenosine-5′-phosphosulfate (APS [37]) by APS reductase encoded by *aprBA* and ATP sulfurylase encoded by *sat*. Obviously, the two alternative pathways may operate depending on the red-ox potential: (i) a sulfide-dependent microoxic pathway at very low red-ox conditions, such as present in the Thiopaq reactor, involving the ‘reversed sulfidogenic’ pathway, and (ii) an aerobic sulfide/thiosulfate oxidation pathway at high red-ox potential, such as in batch cultures with thiosulfate, involving the truncated Sox cluster and SorA. The presence of different copies of genes involved in the sulfur metabolism might indicate the adaptation of this organism to a highly specialized sulfide oxidation lifestyle.

### Energy metabolism

Although we are gaining some insight into the bioenergetics of alkaliphilic heterotrophs, such as *Bacillus* species [38], it is a complete mystery how haloalkaliphilic chemolithoautotrophic bacteria obtain enough energy for growth. To generate NADH for CO_2_ fixation, chemolithoautotrophic bacteria, using inorganic compounds (e.g. H_2_S or NH_3_) as electron donors, have to transport electrons against the thermodynamic gradient (‘reverse electron transport’), which is an energy-requiring process. In addition, those that are living at high salt concentrations, have to invest extra energy in the production of organic compatible solutes. And thirdly, bacteria that live at high pH have to invest additional energy to maintain their pH homeostasis.

So, to obtain enough energy for growth the haloalkaliphilic chemolithoautotrophic SOB must have a special adaptation of their bioenergetics. The most obvious solution would be the presence of primary sodium pumps, such as a sodium-driven ATP synthase, but genes for this could not be detected; instead we found all the genes for a proton-driven F0F1-type ATP synthase (i.e., subunit A, B, and C of the F0 subcomplex, and subunit alpha, beta, gamma, delta, and epsilon of the F1 subcomplex). The presence of a proton-driven ATP synthase instead of a sodium-driven ATP synthase has been found in all genomes of so far studied aerobic alkaliphilic bacteria studied thus far [39]. However, we could detect several genes encoding different sodium-dependent pumps, such as the primary sodium pump Rnf and secondary pumps, such as the Na^+^/H^+^ antiporters NhaP and Mrp, a sodium:sulfate symporter (SulP), and the sodium-depending flagellar motor PomA/B. Apart from the genes encoding the proton-translocating NADH dehydrogenase (*nuoABCDEFGHIJKLMN*), we also found genes (*rnfABCDGE*) that are homologous to the *nqr* genes encoding the sodium-translocating NADH:quinone oxidoreductase (Na^+^-NQR [40], ). Na^+^-NQR was first discovered in the marine bacterium *Vibrio alginolyticus* [41]. It is coupled to the respiratory chain, and oxidizes NADH with ubiquinone as electron acceptor. The free energy released is used to generate a sodium motive force at the FAD-quinone coupling site. The presence of both a proton- and sodium-translocating NADH:quinone oxidoreductase in one organism was described previously by Takada *et al*. [42]. They showed that both pumps were very active in a psychrophilic bacterium, *Vibrio* sp. strain ABE-1, growing at low temperatures. It is, of course, not clear what the role of either pump is in our strain, but it is tempting to speculate that they are a special adaptation to generate enough energy for growth under these extreme conditions. Future transcriptomic and proteomic studies are necessary to validate this speculation. NhaP is a Na^+^/H^+^-antiporter (a secondary sodium pump), which plays a role in the regulation of the internal pH of the cell; it pumps sodium out of the cell and leaves protons and ensuing energy generated by the respiratory chain. Furthermore, we found all 7 genes (*mnhA-G*) for the multisubunit Na^+^/H^+^-antiporter Mrp, which may play a similar role as NhaP. Apart from genes encoding proton-driven flagellar motors (motA/B), we also found genes encoding sodium-driven flagellar motors (pomA/B). Phylogenetic analysis of the motA/B and pomA/B grouped them with sequences of other bacteria, such as *Halorhodospira halophila* and *Alkalilimnicola ehrlichii* (results are not shown).

**Figure 5:**
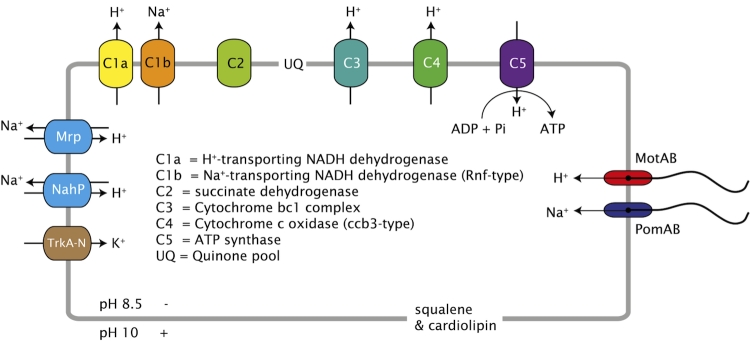
Conceptual model of the different proton and sodium primary pumps, secondary transporters and flagellar motors in *Thioalkalivibrio* “sulfidophilus” HL-EbGr7.

We have also found genes for the production of cardiolipin (cardiolipin synthase) and of squalene (squalene synthase), confirming the high concentrations of these compounds in the cell membranes of another *Thioalkalivibrio* strain, strain ALJ15 [43]. These compounds contribute indirectly to an efficient energy metabolism, as the negatively charged cardiolipids might trap protons at the cell membrane preventing them from diffusing into the environment [44], and squalene lowers the proton permeability of the lipid bilayer [45]. From the genes that we found, we made the following conceptual model (Figure 5).

### Compatible solutes

*Thioalkalivibrio* species are characterized by their tolerance to high salt concentrations, which can be up to 4.3M total sodium [2,17]. To withstand these hypersaline conditions, these species synthesize glycine-betaine as the main compatible solute. In one of the high-salt *Thioalkalivibrio* strains, Banciu et al. [43] showed a positive correlation between salinity and the intracellular glycine-betaine concentration, and found that glycine-betaine constituted 9% of cell dry weight at 4M of sodium in the culture medium. In most cases, betaine is synthesized from choline by a two-step oxidation pathway [46]. However, an alternative route is the synthesis of betaine by a series of methylation reactions [47]. The genome of strain HL-EbGr7 contains genes coding for glycine sarcosine N-methyltransferase and sarcosine dimethylglycine methyltransferase, that are catalyzing betaine synthesis from glycine in a three-step methylation process, i.e., glycine -> sarcosine -> dimethylglycine -> betaine. The sequences of the 2 enzymes have high similarities to sequences found in the close relatives *Halorhodospira halophila* and *Nitrococcus mobilis.* Apart from glycine-betaine *Thioalkalivibrio* species also produce sucrose as a minor compatible solute (up to 2.5% of cell dry weight at 2M of sodium) [43]. The genomes of strain HL-EbGr7 contain genes coding for the enzymes sucrose synthase and sucrose phosphate synthase, which both play a role in the synthesis of sucrose. In contrast to other members of the *Ectothiorhodospiraceae,* i.e., *Alkalilimnicola ehrlichii*, and *Halorhodospira halodurans*, no genes were found for ectoine synthesis in the genome of HL-EbGr7.
